# Preoperative IL-8 levels as prognostic indicators of overall survival: an extended follow-up in a prospective cohort with colorectal liver metastases

**DOI:** 10.1186/s12885-023-11787-1

**Published:** 2024-01-17

**Authors:** Mathieu Pecqueux, Frederik Brückner, Florian Oehme, Sebastian Hempel, Franziska Baenke, Carina Riediger, Marius Distler, Jürgen Weitz, Christoph Kahlert

**Affiliations:** 1 Department of Visceral, Thoracic and Vascular Surgery, Faculty of Medicine and University Hospital Carl Gustav Carus Technische Universität Dresden, Fetscherstrasse 74, 01307 Dresden, Germany; 2 National Center for Tumor Diseases (NCT/UCC), Dresden, Germany: German Cancer Research Center (DKFZ), Heidelberg, Germany; Faculty of Medicine and University Hospital Carl Gustav Carus, Technische Universität Dresden, Dresden, Germany; Helmholtz-Zentrum Dresden - Rossendorf (HZDR), Dresden, Germany

**Keywords:** Colorectal cancer, Prognostic biomarker, Interleukin-8, IL-8

## Abstract

**Introduction:**

CRC with liver metastases is a major contributor to cancer-related mortality. Despite advancements in liver resection techniques, patient survival remains a concern due to high recurrence rates. This study seeks to uncover prognostic biomarkers that predict overall survival in patients undergoing curative hepatic resection for CRC liver metastases.

**Methods:**

Prospectively collected serum samples from a cohort of 49 patients who received curative hepatic resection for CRC liver metastases were studied. The patients are part of a cohort, previously analyzed for perioperative complications (see methods). Various preoperative serum markers, clinical characteristics, and factors were analyzed. Univariate and multivariate Cox regression analyses were conducted to determine associations between these variables and disease-free survival as well as overall survival.

**Results:**

For disease-free survival, univariate analysis highlighted the correlation between poor outcomes and advanced primary tumor stage, high ASA score, and synchronous liver metastases. Multivariate analysis identified nodal-positive primary tumors and synchronous metastases as independent risk factors for disease-free survival. Regarding overall survival, univariate analysis demonstrated significant links between poor survival and high preoperative IL-8 levels, elevated neutrophil–lymphocyte ratio (NLR), and presence of metastases in other organs. Multivariate analysis confirmed preoperative IL-8 and having three or more liver metastases as independent risk factors for overall survival. The impact of IL-8 on survival was particularly noteworthy, surpassing the influence of established clinical factors.

**Conclusion:**

This study establishes preoperative IL-8 levels as a potential prognostic biomarker for overall survival in patients undergoing curative liver resection for CRC liver metastases. This study underscores the importance of incorporating IL-8 and other biomarkers into clinical decision-making, facilitating improved patient stratification and tailored treatment approaches. Further research and validation studies are needed to solidify the clinical utility of IL-8 as a prognostic marker.

## Introduction

Colorectal cancer (CRC) ranks as the third most commonly diagnosed cancer ( 10%) and the second leading cause of cancer-related mortality (9.4%) worldwide [1]. Colorectal liver metastases are observed in approximately 30–50% of all CRC cases and represent the primary cause of mortality [2, 3]. The ongoing progress in diagnostic and treatment methods has broadened the range of cases and the effectiveness of liver resections [4], solidifying it as a well-established curative choice, especially for treating colorectal cancer metastases in the liver. Consequently, there has been a substantial increase in the success rates for 5- and 10-year survival among patients who undergo curative R0 resections for metastatic colorectal cancer, which has prompted a greater frequency of and more extensive hepatic resections [5]. However, despite these improvements, up to 90% of patients experience cancer recurrence within 3 years, with 50% recurrence within the first year after resection [6]. In this context, re-resections are frequently performed and can offer prolonged survival [7]. Nonetheless, the overall survival for metastatic disease remains moderate, with a median 5 and 10-year survival rates of 38% and 26%, respectively [5]. To address this challenge, identifying prognostic risk factors for survival has been the subject of research efforts [6, 8]. The discovery of novel biomarkers may enhance the prediction of survival of patients with metastatic hepatic cancer and could potentially influence clinical decision-making.

Inflammation is recognized as a critical factor in cancer progressio*n and metastasis. Inflammatory scoring systems, such as the modified Glasgow Prognostic* Score (mGPS), which includes C-reactive protein (CRP) and Albumin, have demonstrated prognostic capability for overall survival in patients with colorectal liver metastases, with CRP emerging as a particularly important prognostic factor [9, 10]. Proinflammatory cytokines, such as interleukin-6 (IL-6) and interleukin-8 (IL-8), are not only closely linked to inflammation but also play pivotal roles in liver disease and regeneration [11, 12]. IL-6, secreted by T lymphocytes, endothelial cells and macrophages, enters the liver through the bloodstream and triggers the acute phase response (APR), leading to CRP production in the liver. On the other hand, IL-8 is produced by macrophages, epithelial cells, endothelial cells, and fibroblasts and it initiates the recruitment of neutrophils.

Recent meta-analyses have highlighted the significance of certain prognostic biomarkers in predicting survival outcomes for patients with colorectal liver metastases. For instance, a high pretreatment neutrophil–lymphocyte ratio (NLR) was associated with significantly poorer survival [13]. Additionally, IL-8 has been implicated as a valuable prognostic marker in colorectal cancer [14], with several studies analyzing its expression in the serum of colorectal cancer patients as a biomarker for survival [15–18]. Among these studies, two include patients receiving palliative chemotherapy [15, 17] and one includes non-metastatic colorectal cancer prior to surgery [18]. Only one study analyzed serum IL-8 prior to resection of colorectal liver metastases using a multiplexed protein bead assay under experimental conditions [16]. However, to the best of our knowledge, our study represents the first prospective analysis of preoperative IL-6 and IL-8 levels under clinical conditions using normal serum tubes within the standard preoperative care for establishing IL-8 as a biomarker in metastatic CRC patients undergoing curative liver resection. Upon rigorous examination of IL-8's potential as a predictive marker for immediate postoperative complications within this cohort of our study [19], our present objective is to discern the prognostic capabilities of IL-8 in conjunction with other parameters, notably the neutrophil–lymphocyte ratio, for long-term outcomes. Such insights would enable the stratification of patients into distinct preoperative risk tiers, facilitating a more tailored postoperative therapeutic approach.

## Methods

### Patient cohort

The patients included in the study are part of the prospectively recruited cohort of patients who underwent hepatic resection at the Department of Surgery, University Hospital Carl Gustav Carus, Dresden, between August 2017, and December 2018. A previous publication investigated the predictive value of pre- and postoperatively assessed inflammatory markers for the detection of postoperative complications [19]. The current study evaluates the prognostic value of preoperative inflammation markers for predicting long time survival in patients who underwent curative hepatic resection for colorectal liver metastases. The study received approval from the Ethics Committee of the University of Dresden (EK290072017). A total of 50 patients received curative hepatic resections for colorectal liver metastases and received preoperative blood sampling including inflammation markers. One patient was excluded from the study due to irresectability. Two cases only included preoperative IL-6 values analysis due to an error in the blood analysis request form and were excluded from IL-8 analyses. All patients provided written informed consent prior to enrollment in the study.

### Operation

All patients underwent preoperative abdominal computed tomography (CT) scans to assess resectability and the extent of the resection, along with a chest CT scan to exclude pulmonary metastases. For patients with an estimated remaining liver volume below 30% or below 40–50% in patients with pre-existing conditions like cirrhosis, cholestasis, fibrosis or prolonged neoadjuvant chemotherapy, preoperative portal vein embolization was performed to increase the volume of the remnant liver tissue. Standardized preoperative, perioperative and postoperative care were provided.

Liver resections were carried out using the crush-clamp technique in combination with diathermy or LigaSure (laparoscopic approach). The Pringle maneuver was only employed in cases of bleeding. Resection of liver tissue was performed at the surgeon’s discretion while maintaining low central vein pressure (< 5 mmHg).

### Serum marker analyses and their prognostic value

Serum samples were collected and analyzed preoperatively within 14 days of resection. The serum panel included standard markers such as leucocytes (GPt/L), platelet count (GPt/L), C-reactive protein (CRP, mg/L), bilirubin (µmol/L), and international normalized ratio (INR). A differential blood count was conducted to calculate the neutrophil–lymphocyte ratio (NLR) and the thrombocyte-lymphocyte ratio (TLR). Additionally, interleukin-6 (IL-6) and interleukin-8 (IL-8) were measured as part of the standardized laboratory diagnostics. The analysis was performed using EDTA plasma and the Immulite 1000 device (Siemens), which employs standardized enzyme-amplified chemiluminescence with assay-specific coated beads. The Immulite 1000 device is approved for in vitro diagnostics of IL-6 and IL-8 in Germany and offers a cost and time-effective option (approximately 10–15 Euros per test) to include these biomarkers in routine diagnostics for specific use cases, such as metastasized colorectal cancer.

### Statistics

Statistical analyses were conducted using SPSS (IBM, version 26). Nominal variables were dichotomized into two categories, with the ASA score dichotomized into a low-risk group (ASA 1–2) and a high-risk group (ASA 3–4) for survival analysis. Numeric scale variables were dichotomized at the median. Survival curves were constructed using Kaplan–Meier methodology, and p-values were calculated using the log-rank test. A significance level of *p* < 0.05 were considered statistically significant. Hazard ratios were determined through univariate and multivariate Cox regression analyses.

## Results

### Patient characteristics

A total of 49 patients who underwent curative hepatic resection for colorectal liver metastases were included in the study, with 19 (39%) females and 10 (61%) males, and a mean age of 60 years (ranging from 28 to 82 years). The majority of patients (69%, *n* = 34) had an American Society of Anesthesiologists (ASA) score of 3, 29% (*n* = 14) a score of 2 and only 2% (*n* = 1) had a score of 1. Neoadjuvant chemotherapy was administered to 47% (*n* = 23) of patients before resection. Adjuvant chemotherapy was administered in 73% (*n* = 36) of patients following resection. The surgical approach was predominantly open surgery (90%, *n* = 44), with laparoscopic resections performed in 10% (*n* = 5) of cases. Wedge resections were conducted in 31% (*n* = 15), one or two segment resection in 41% (*n* = 20), and hemihepatectomy in 29% (*n* = 14). Microscopic margin-free resection (R0) was achieved in 90% (*n* = 44) of cases, while 4% (*n* = 2) had microscopic tumor residue, 4% (*n* = 2) had macroscopic tumor residue, and 2% (*n* = 1) could not be evaluated (Rx). Tumor grade assessment was not possible in 45% (*n* = 22) of patients due to neoadjuvant chemotherapy. Among the remaining patients, most had tumor grade G2 (*n* = 19), followed by G3 (*n* = 7), and one patient with G1. During the follow up, 80% of patients experienced relapse, and 57% died. The median follow-up for the remaining patients was 36 months. Additional details can be found in Table 1.

**Table 1:** Clinical data of the patients included in the study as well as for patients with low or high preoperative IL-8 values

	**All (*n* = 49)**	**Low preoperative IL-8 (IL-8 < 10; *n* = 23)**	**High preoperative IL-8 (IL-8 ≥ 10; *n* = 24)**	***P* value**
**Age (mean + range)**	60 (28–82)	58 (28–79)	61 (35–82)	0.41
**Sex f/m (f%/m%)**	19/30 (39%/61%)	9/14 (39%/61%)	10/14 (42%/58%)	0.53
**ASA**				
**I**	1 (2%)	1 (4%)	0 (0%)	0.86
**II**	14 (29%)	6 (26%)	8 (33%)	
**III**	34 (69%)	16 (70%)	16 (67%)	
**Tumor stage**				
**T1**	1 (2%)	1 (4%)	0 (0%)	0.69
**T2**	9 (18%)	4 (17%)	4 (17%)	
**T3**	34 (69%)	15 (65%)	18 (78%)	
**T4**	5 (10%)	3 (13%)	2 (9%)	
**Lymph nodes**				
**N0**	20 (41%)	10 (43%)	10 (43%)	0.84
**N1**	15 (31%)	7 (30%)	6 (26%)	
**N2**	14 (29%)	6 (26%)	8(35%)	
**Grading**				
**G1**	1 (2%)	1 (4%)	0 (0%)	0.31
**G2**	19 (39%)	11 (48%)	7(30%)	
**G3**	7 (14%)	2 (9%)	5 (22%)	
**Gx**	22 (45%)	9 (39%)	12 (52%)	
**Resection**				
**R0**	44 (90%)	21 (91%)	21 (88%)	0.40
**R1**	2 (4%)	1 (4%)	1 (4%)	
**R2**	2 (4%)	0	2 (8%)	
**Rx**	1 (2%)	1 (4%)	0	
**Synchronous Metastases**	34 (69%)	15 (65%)	18 (75%)	0.52
**Open surgery**	44 (90%)	19 (83%)	23 (96%)	0.14
**Laparoscopic surgery**	5 (10%)	4 (17%)	1 (4%)	0.14
**Resection**				0.75
**Wedge**	15 (31%)	6 (26%)	7 (29%)	
**Segment**	20 (41%)	10 (43%)	10 (42%)	
**HH**	14 (29%)	7 (30%)	7 (29%)	
**Neoadj.Chemo**	23 (47%)	11 (48%)	10 (42%)	0.87
**No complication**	31 (63%)	8 (35%)	9 (37%)	0.85
**Complications**	18 (37%)	15 (65%)	15 (63%)	0.85
**Clavien Dindo**				0.43
**CD I**	2 (4%)	0	2 (4%)	
**CD II**	6 (12%)	4 (17%)	2 (12%)	
**CD IIIa**	6 (12%)	3 (13%)	2 (8%)	
**CD IIIb**	4 (8%)	1 (4%)	3 (8%)	
**CD IV**	0 (0%)	0 (0%)	0 (0%)	
**CD V**	0 (0%)	0 (0%)	0 (0%)	
**Days ICU mean (min–max)**	1.4 (0–10)	1.4 (0–6)	1.5 (0–10)	0.83
**Days hospitalized (min–max)**	10.3 (3–49)	11.2 (3–49)	9.5 (4–28)	0.98
**Median follow up (months)**	36	36	24	
**Relapsed during follow up**	39 (80%)	18 (78%)	20 (83%)	0.66
**Died during follow up**	28 (57%)	11 (48%)	16 (67%)	0.19
**CEA > 60**	6	3	3	0.73
**Other Mets than liver**	16	6	10	0.26
**Largest Met > 4 cm**	14	6	8	0.59
**≥ 3 Livermets**	27	13	14	0.90

ASA indicates the American Society of Anesthesiologists score; HH indicates hemihepatectomy; Segment indicates the resection of 1–2 segments; ICU indicates intensive care unit stay

### Univariate survival analysis

The primary objective of this study was to evaluate the prognostic capacity of preoperative markers as prognostic markers for overall survival in patients undergoing liver surgery for colorectal cancer. Preoperative serum markers were dichotomized based on the median and their correlation with overall survival was examined. Moreover, various clinical factors, such as preoperative ASA score, diabetes prevalence, neoadjuvant chemotherapy, previous liver resections, number of metastases, size of the largest metastasis, age, BMI, extent of the liver resection (wedge to two segments vs. hemihepatectomy), and extent of the primary tumor (Tumor stage and lymph node stage) were also analyzed.

Univariate Cox regression analyses revealed significant correlations with disease-free survival for patients with synchronous metastases (HR 3.33; 95%CI 1.10 – 10.14; *p* = 0.03), advanced T stage (HR 3.47; 95%CI 1.16 – 10.39; *p* = 0.03) and higher ASA score (HR 2.42; 95%CI 1.05 – 5.57; *p* = 0.04) (Table 2). Subsequent multivariate Cox regression, including factors with p-values < 0.2 in the univariate analyses, identified only positive lymph nodes and synchronous liver metastasis to be as independent factors associated with adverse disease-free survival (Table 3). Among these factors, only synchronous metastasis demonstrated a significant association with disease-free survival, while the positive lymph node status did not reach statistical significance (*p* = 0.056).

**Table 2:** Univariate Cox regression analysis for disease free survival

Univariate Cox Regression analysis for Recurrence free survival			
	**Hazard**	**95% CI interval**	***p*-Value**
**Leuk**	1.19	0.55—2.57	0.66
**NLR**	0.58	0.26—1.31	0.19
**TLR**	0.57	0.26—1.29	0.18
**CRP**	1.35	0.63—2.90	0.44
**IL6**	0.49	0.22—1.08	0.08
**IL8**	1.06	0.50—2.25	0.87
**Bilirubin**	1.42	0.66—3.03	0.37
**Quick**	0.69	0.32—1.47	0.33
**Sex**	0.92	0.43—1.97	0.83
**Synchronous metastasis**	3.33	1.10—10.14	0.03
**N pos**	2.09	0.90—4.87	0.09
**T stage ≥ 3**	3.47	1.16—10.39	0.03
**G3**	0.48	0.13—1.76	0.27
**ASA ≥ 3**	2.42	1.05—5.57	0.04
**Diabetes**	0.62	0.14—2.69	0.52
**neoadj. CTX**	1.16	0.85—1.59	0.36
**No previous liver resections**	1.18	0.56—2.51	0.67
**3 or more Metastases**	1.76	0.78—3.93	0.17
**other Metastases**	0.78	0.31—.99	0.61
**largest Liver-Metastasis > = 4 cm**	0.87	0.39—1.93	0.73
**CEA > 60**	0.92	0.34—2.49	0.86
**R0**	1.53	0.52—4.53	0.44
**Age > 70**	1.03	0.48—2.20	0.95
**BMI larger 30**	1.55	0.53—4.52	0.43
**Major liver resection**	1.05	0.47—2.33	0.91

NL = Neutrophil Lymphocyte Ratio; TL = Thrombocyte Lymphocyte ratio

*CRP* C-reactive Protein, *IL-6* Interleukin 6,m *IL-8* Interleukin 8, *Bili* bilirubin, CEA

**Table 3:** Multivariate Cox regression for disease free survival including all factors with a *p*-value < 0.2 in the univariate analysis

Multivariate cox regression analysis for Disease free survival			
	**Hazard**	**95% CI interval**	***p*-Value**
**Positive Lymph nodes**	2.65	0.98 – 7.22	0.056
**Synchronous metastasis**	4.32	1.24 – 15.09	0.022

Multivariate Cox regression analysis was performed by forward stepwise selection including all factors with *p* ≤ 0.2 in the univariate Cox regression analysis

Regarding overall survival, univariate Cox regression analyses demonstrated that preoperative Interleukin-8 levels ≥ 10 (HR 2.23; 95%CI 1.03 – 4.83; *p* = 0.04), preoperative neutrophil–lymphocyte ratio (NLR) (HR 2.61; 95%CI 1.12 – 6.08; *p* = 0.03), and the presence of metastases in organs other than the liver (HR 2.42; 95%CI 1.13 – 5.19; *p* = 0.02) were significantly correlated with poor overall survival. Preoperatively increased Interleukin 6 (IL-6) showed a trend towards worse survival (HR 2.13 95%CI 0.97 – 4.68; *p* = 0.06), as did synchronous liver metastases (HR 2.5; 95% CI 0.93 – 6.71; *p* = 0.07). Further details are available in Table 4.

**Table 4:** Univariate Survival analysis for overall survival (Univariate Cox regression; shown are the Hazard ratio, the 95% confidence interval and the *p*-value. *P*-values < 0.05 were regarded as significant)

Univariate Cox Regression analysis for Overall Survival			
	**Hazard**	**95% CI interval**	***p*-Value**
**Leuk**	0.9	0.42—1.19	0.76
**NLR**	2.61	1.12—6.08	0.03
**TLR**	1.17	0.53—2.58	0.7
**CRP**	1.69	0.77—3.69	0.19
**IL6**	2.13	0.97—4.68	0.06
**IL8**	2.23	1.03—4.83	0.04
**Bilirubin**	1.44	0.68—3.06	0.35
**Quick**	0.71	0.34—1.49	0.36
**Sex**	0.6	0.26—1.37	0.23
**Synchronous metastasis**	2.5	0.93—6.71	0.07
**N pos**	1.86	0.84—4.14	0.13
**T stage ≥ 3**	1.94	0.72—5.19	0.19
**G3**	1.75	0.56—5.43	0.34
**ASA ≥ 3**	1.43	0.65—3.14	0.37
**Diabetes**	0.38	0.07—2.19	0.28
**neoadj. CTX**	1.02	0.76—1.38	0.89
**No previous liver resections**	0.57	0.27—1.21	0.14
**3 or more Metastases**	1.92	0.84—4.36	0.12
**other Metastases**	2.42	1.13—5.19	0.02
**largest Liver-Metastasis > = 4 cm**	1.25	0.56—2.77	0.59
**CEA > 60**	0.95	0.36—2.56	0.93
**R0**	1.04	0.31—3.18	0.94
**Age > 70**	1.15	0.54—2.46	0.72
**BMI larger 30**	0.69	0.21—2.29	0.54
**Major liver resection**	0.6	0.24—1.48	0.27

NL = Neutrophil Lymphocyte Ratio; TL = Thrombocyte Lymphocyte ratio

*CRP* C-reactive Protein, *IL-6* Interleukin 6, *IL-8* Interleukin 8, *CEA*

These results indicate that the preoperative Interleukin 8 levels, preoperative neutrophil–lymphocyte ratio (NLR), and metastases in other organs besides the liver can predict postoperative overall survival.

### Multivariate survival analysis

To assess the independent impact of preoperative variables on overall survival, a multivariate Cox regression analysis was conducted, including all factors with p-values < 0.2 in the univariate Cox regression analyses. The multivariate analysis identified preoperative interleukin-8 (HR 3.30; 95% CI 1.37 – 8.25; *p* = 0.008) and the presence of more than three liver metastases (HR 2.85; 95% CI 1.12–7.93; *p* = 0.03) as independent risk factors for poor overall survival (Table 5).

**Table 5:** Multivariate Cox regression for overall survival including all factors with a *p*-value < 0.2 in the univariate analysis

Multivariate cox regression analysis for Overall Survival			
	**Hazard**	**95% CI interval**	***p*-Value**
**Preoperative IL-8**	3.30	1.37 – 8.25	0.008
**3 or more Metastases**	2.85	1.12 – 7.93	0.029

Multivariate Cox regression analysis was performed by forward stepwise selection including all factors with *p* ≤ 0.2 in the univariate Cox regression analysis

**Fig. 1:**
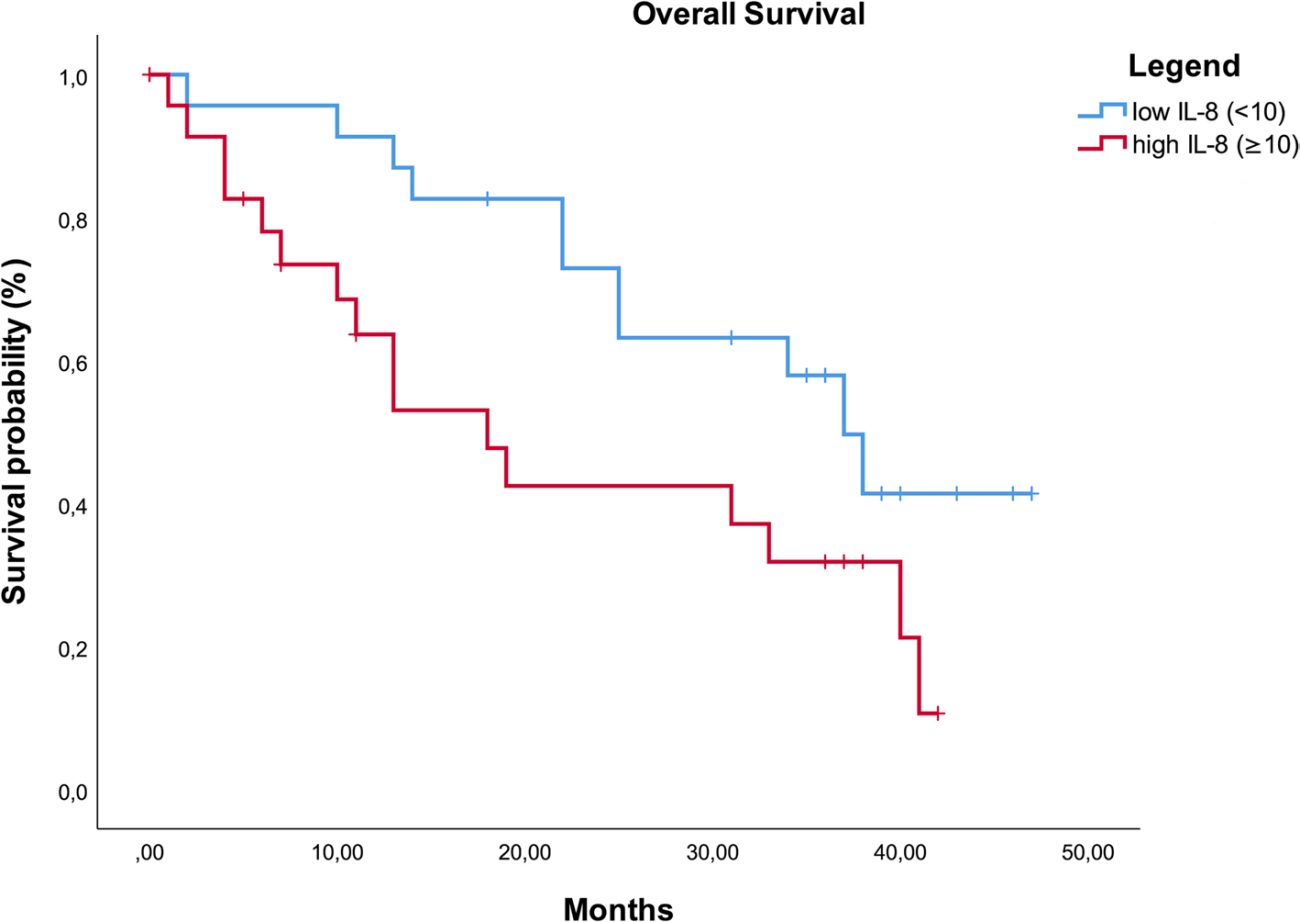
Kaplan Meier curve for Overall survival in patients with low (< 10) or high (≥ 10)preoperative Interleukin 8 (IL-8) values. Log Rank test: *p* = 0.035)

Additionally, to evaluate the potential of preoperative IL-8 as a marker for overall survival in patients undergoing liver resection for colorectal liver metastases, the clinical factors in the groups with low (< 10) and high (≥ 10) IL-8 levels were compared, showing no significant differences (Table 1). Kaplan–Meier curve analysis depicted overall survival in patients with low and high IL-8 levels revealing a steady divergence of the survival curves until 24 months, after which both curves remained parallel to each other (Fig. 1).

In order to assess the impact of neoadjuvant chemotherapy, a recognized therapeutic intervention with known effects on intratumoral immunity [20], we compared preoperative IL-6 and IL-8 levels between patients who underwent neoadjuvant chemotherapy and those wo did not. Our findings indicate no meaningful difference in the levels of IL-6 and IL-8 between the two groups. (IL-6: *p* = 0.84; IL-8 *p* = 0.90). Additionally, upon comparing patients with high and low preoperative IL-8 levels, no significant differences were observed in any clinical factors, including the administration of neoadjuvant chemotherapy.

### Limitations of the study

Limitations of the study include its single-center nature, which introduces the possibility of bias inherent to a specific patient population. Furthermore, while the testing equipment used is certified for the detection of IL-8, not every hospital has IL-8 detection in its standard repertoire. Strong points of the study are the prospective nature of the data acquisition, minimizing selection bias, as well as the relatively high number of included patients (*n* = 49).

While the certified testing equipment for IL-8 detection may not be available in all hospitals, the standardized and certified testing equipment greatly improves the reliability and validity of the results. Furthermore, the study acknowledges the cost of associated with measuring IL-8 (approx. 10–15€ per test). While this cost may be a practical consideration, this could play an essential role in the later adaptation into clinical practice.

## Discussion

With advancements in surgical techniques, liver resections for colorectal cancer have become safer, with median 5 and 10 year survival rates of 38% and 26% respectively [5]. In cases of recurrence, repeated resections are feasible. Various clinical risk scores are used to stratify the risk of recurrence and survival, aiding in determining the optimal treatment approach: either curative resection or systemic therapy. These scores primarily rely on clinical factors such as the number and size of the liver metastases, nodal status of the primary tumor, disease-free interval, and serum markers like preoperative CEA [6, 8, 21–26]. Additionally, inflammation-based scores like the modified Glasgow prognostic score (mGPS), focus on C-reactive protein (CRP) [10]. The liver’s close association with inflammation and immunity enables hepatocytes to play a direct or indirect role by secreting bactericidal proteins and acute phase proteins such as IL-6 and IL-1β [27–29]. Interleukin 6 is produced by immune cells, epithelial cells, and hepatocytes themselves. In response, hepatocytes produce acute phase proteins, especially CRP [29].

Another inflammatory protein, IL-8, has been linked to chronic liver inflammation, hepatic macrophage accumulation and liver fibrosis [12]. It acts as a potent chemoattractant for neutrophils and has been associated with poor overall survival in colorectal cancer [15–18]. While surgery remains the only curative option in metastatic colorectal cancer, the continuous progress in the field has made it a safe option with reasonable 5 year overall survival rates. The challenge lies in identifying patients who would benefit most from surgery or systemic therapy prior to surgery. One study has explored comprehensive serum inflammation panels using multiplexed protein bead assays prior to curative surgery. Despite the exploratory nature of this study and the relatively low number of patients analyzed compared to the high number of inflammation proteins, they identified independent markers of poor overall survival, including Interleukin 8, PDGF-AB/BB, eotaxin and IP-10.

In this study, we investigated the potential of preoperative inflammatory serum markers, including Interleukin-6 (IL-6), C-reactive protein (CRP) and Interleukin-8 (IL-8), as biomarkers for long-termn survival in patients with curatively resectable colorectal cancer (CRC) and liver metastases. In our prior analysis of this cohort during short-term follow-up, IL-8 demonstrated superior prognostic value compared to CRP for severe complications in patients undergoing extensive hepatic surgical interventions [19]. Other inflammatory markers such NLR and TLR, along with well-established clinical factors were included for comparison. The results of the univariate analyses for disease-free survival showed significant correlation with advanced primary tumor stage (T-stage), poor ASA score, and synchronous liver metastases. There was also a trend for IL-6 and nodal positive primary tumor. Multivariate analysis showed only nodal positive primary tumor and synchronous metastasis to be independent risk factors, with synchronous metastasis being the only significant independent risk factor. However, the data quality for disease-free survival was challenging to assess due to follow-up intervals and multiple oncologists involved.

Regarding overall survival, the univariate analysis identified the NLR, IL-8, and metastases outside the liver as significant survival factors. IL-6 showed a trend, while CRP and CEA did not correlate significantly with survival. The multivariate analysis revealed that IL-8 and the presence of three or more liver metastases were independent risk factors, with IL-8 having the most significant impact. Comparing patients with high or low IL-8 shows no significant differences in the clinical parameters, confirming that IL-8 is indeed an independent risk factor.

IL-8, also known as CXCL8, is a proinflammatory chemokine produced by various cell types, including macrophages, epithelial cells, endothelial cells and fibroblasts. It primarily binds to CXCR1 and CXCR2 receptors expressed on monocytes, granulocytes, endothelial cells and tumor-associated macrophages, leading to neutrophil recruitment at sites of inflammation.

Cancer cells often overexpress IL-8 to modify the tumor microenvironment. IL-8 is closely linked to cancer metabolism, inflammation and progression, functioning through autocrine signaling and angiogenic stimulation of endothelial cells and can represent tumor mass [30–32]. Studies have shown that high fat diet can influence the inflammatory microenvironment in mice models via IL-8, leading to accelerated tumor development in Barrett’s esophagus [33]. Furthermore IL-8 has been implicated in promoting cancer cell emergence from dormancy in breast cancer [34].

In diverse malignancies, IL-8 has been implicated in enhancing invasiveness, metastasis, and resistance to chemotherapy [35–37]. Additionally, elevated serum IL-8 levels have been correlated with resistance to anti-PD-1 therapy in melanoma and non-small- cell lung cancer [38, 39]. This is consistent with the observed relationship between serum IL-8 levels and poor survival in various cancer types, including pancreatic cancer [40], breast cancer [41], and colorectal cancer [14]. Notably, a meta-analysis demonstrated a significant association between IL-8 and overall survival in colorectal cancer [14]. Although all studies but one were retrospective and used varied detection methods (qPCR, Immunohistochemistry, ELISA and Multiplex) on patients with either metastasized or primary cancer, and most analyses were conducted on cancer tissue, the evidence points towards a clear relevance of IL-8 for the overall survival of colorectal cancer patients. Furthermore, liver diseases such as nonalcoholic steatohepatitis (NASH) have been linked to elevated IL-8 levels, providing further support for the role of IL-8 levels in inflammation, particularly within the liver [42].

Contrary to expectations, our study did not reveal significant differences in the proportion of patients undergoing neoadjuvant chemotherapy between the IL-8 high and low groups. Furthermore, there were no notable variations in IL-6 or IL-8 serum levels between patients who underwent neoadjuvant chemotherapy and those who did not. These findings suggest that neoadjuvant chemotherapy did not exert a discernible influence on the prognostic capacity of preoperative IL-8 levels.

While IL-8 emerged as a significant independent prognostic factor for overall survival, its prognostic value for recurrence-free survival was not evident. This observation aligns with one study included in the meta-analysis on the relevance of IL-8 in colorectal cancer that mentions progression-free survival [17]. This discrepancy may be attributed to the data quality for recurrence-free survival being weaker than for overall survival. The relatively swift recurrence observed in most patients in our study, with a median recurrence after 5 months, poses challenges in discerning differences, particularly considering the average follow-up interval of 3–6 months. Moreover, we demonstrated that preoperative IL-8 is significantly associated with the occurrence of major postoperative complications after liver surgery [19]. Postoperative complications can impact overall survival through inflammatory conditions conducive to tumor growth and by causing delays in adjuvant therapy [43]. These factors could explain the superior prognostic value of IL-8 on overall survival.

The combination of factors such a predisposition for major complications, increased inflammation, association with liver disease, increased tumor aggressiveness, and resistance to chemotherapy, could collectively explain the robust prognostic value of IL-8 levels for overall survival in this study.

## Conclusion

In conclusion, this study is the first to demonstrate that IL-8 can be routinely assessed using standardized methods during preoperative blood sampling and may serve as a valuable tool to predict overall survival in patients undergoing liver surgery for metastasized colorectal cancer. The strong prognostic impact of IL-8 in comparison to known clinical factors suggests its potential as a significant prognostic marker in this setting. However, further research and validation studies are needed to fully establish its clinical utility in routine practice.

## Data Availability

The datasets generated and analyzed during the current study are not publicly available but are available from the corresponding author on reasonable request.
